# Overweight and diabetes prevention: is a low-carbohydrate–high-fat diet recommendable?

**DOI:** 10.1007/s00394-018-1636-y

**Published:** 2018-03-14

**Authors:** Fred Brouns

**Affiliations:** Department of Human biology, Faculty of Health, Medicine and Life Sciences, NUTRIM-School of Nutrition and Translational Research in Metabolism, Maastricht University, Post Box 616, 6200 MD Maastricht, The Netherlands

**Keywords:** Low-carbohydrate diet, High-fat diet, Ketogenic diet, Type 2 diabetes, Obesity.

## Abstract

In the past, different types of diet with a generally low-carbohydrate content (< 50–< 20 g/day) have been promoted, for weight loss and diabetes, and the effectiveness of a very low dietary carbohydrate content has always been a matter of debate. A significant reduction in the amount of carbohydrates in the diet is usually accompanied by an increase in the amount of fat and to a lesser extent, also protein. Accordingly, using the term “low carb–high fat” (LCHF) diet is most appropriate. Low/very low intakes of carbohydrate food sources may impact on overall diet quality and long-term effects of such drastic diet changes remain at present unknown. This narrative review highlights recent metabolic and clinical outcomes of studies as well as practical feasibility of low LCHF diets. A few relevant observations are as follows: (1) any diet type resulting in reduced energy intake will result in weight loss and related favorable metabolic and functional changes; (2) short-term LCHF studies show both favorable and less desirable effects; (3) sustained adherence to a ketogenic LCHF diet appears to be difficult. A non-ketogenic diet supplying 100–150 g carbohydrate/day, under good control, may be more practical. (4) There is lack of data supporting long-term efficacy, safety and health benefits of LCHF diets. Any recommendation should be judged in this light. (5) Lifestyle intervention in people at high risk of developing type 2 diabetes, while maintaining a relative carbohydrate-rich diet, results in long-term prevention of progression to type 2 diabetes and is generally seen as safe.

## Introduction

The World Health Organisation (WHO) and various national authorities have recently made recommendations urging a limitation of the daily consumption of carbohydrates, more specifically that of rapidly digestible starches and sugars. These recommendations play a key role in reducing the risks of obesity, diabetes, and cardiovascular diseases [1–8]. In the past, there have been various diets that centre on a low-carbohydrate content, such as the Atkins Diet, the Zone Diet, the South Beach Diet and the ketogenic diet [9–13].

In this respect, it should be pointed out that although many studies refer explicitely to “diets with a low-carbohydrate content”, this in fact goes hand in hand with “elevated fat”. This well-known “seesaw effect” is present in many nutritional intervention studies in which a decrease of one particular component always is accompanied by a parallel increase of another component. In other words, effects observed are then based on two dietary factors that were changed in parallel and any conclusion drawn should be viewed in this light. Accordingly the term “low carbohydrate–high fat diet” (LCHF diet) is more appropriate than “low carbohydrate” alone, also in terms of interpretation of results. For this reason, the term LCHF will be used throughout this paper.

Feinman et al. [14] proposed that dietary carbohydrate restriction is the first approach in diabetes management, the authors refer to much data showing that favorable effects such as improvement of insulin sensitivity/needs occurred along with significant weight loss. However, many of the cited studies concerned relatively small groups of individuals, often with poor diet adherence and relatively high dropout rates. Accordingly, one may question the validity of their proposal, especially since recent meta-analyses, which did include well-controlled studies did not see beneficial changes in many parameters on the longer term (this will be discussed further in detail below). Important questions that ought to be put in this respect include: (a) what are the real long-term effects of very low-carbohydrate, thus high-fat intake; (b) are the effects observed rather the result of weight loss than of low carbohydrate per se, and (c) are there less drastic alternatives, i.e. more moderate changes of carbohydrate and fat intake that are easier to adhere to and lead to similar favorable results. These questions are also relevant, given that most chronic diseases such as diabetes, cardiovascular diseases and other chronic conditions have a development period of 10–20 years or more. Long-term data, demonstrating favorable effects of a (very) LCHF diet in this regard, is absent. In addition, there is also a plethora of studies that show that maintaining a relatively high-carbohydrate, low-glycemic–high-fiber diet (vegetarian, vegan) results in favorable long-term effects. Such diet patterns deviate less drastically from our normal eating patterns and are easier to implement in the long term [15–21]. In this respect, observations made in the so-called “blue zones”, e.g. Sardinia, Okinawa (Japan), Loma-Linda (California), are intriguing, given their commonality is their relatively high-carbohydrate and low-saturated fat content of their daily diet, which allow them to stay healthy until very high age. The Okinawa inhabitants, for example, originally ate a daily diet that contained an amount of carbohydrates that exceeded daily energy intake by 60%, consisting primarily of sweet potatoes and the foliage, supplemented with seaweed and fruit. The recent introduction of a more Western lifestyle, containing more saturated fats, added sugars and alcohol, has gone to the significant detriment of the longevity prospects of the youngest generation [22], which indicates that diet (carbohydrate and fat quality), in combination with other lifestyle factors is crucial for health.

An important question is why a challenging LCHF diet, with risks of poor adherence, should be implemented when less drastic changes in diet and lifestyle have proven effects and are known to be safe and easier to follow on the long run. Opinions seem to be sharply divided on the matter. This narrative review will shine a light on the various international opinions on the matter.

## Is a high carbohydrate content of the diet unhealthy?

There are various publications that assert that high levels of carbohydrates are unhealthy. The arguments to support this are often based on assumptions on how man was to have eaten long ago, before the agricultural and industrial revolution. This is referred to as the Paleo Diet. Some authors explicitly claim that our ancestors ate a diet high in fat and protein and that starches and cereals were not part of the daily diet [23]. Historical data available to us, however, have shown that the diet 50,000 years ago, in fact, was relatively high in carbohydrates, that it contained a high level of fiber (from plant-based foods) and that the level of fats primarily depended on the fat content of the various types of meat and fish available [24]. The Paleo diet contained an estimate of ≈ 35 percent of energy (en%) from fats, ≈ 35 en% from carbohydrates and ≈ 30 en% from proteins, with approx. 100 g of dietary fiber a day [24–26]. Consequently, in quantitative terms, the Paleo diet contained roughly as much fat as does our modern Western diet. Observations made by Kaplan et al. [27] who studied the Tsimane population in South America are of great interest in this respect. This population lives a traditional hunter–gatherer lifestyle and ingests an estimated 14% of their average caloric diet as protein, 14% as fat, and 72% as carbohydrate. Yet, despite this very high-carbohydrate intake, the Tsimane have the lowest reported levels of chronic disease of any population ever recorded to date! Today, there are no arguments to suggest that the diet of our ancestors was low in carbohydrate. Quality but not quantity of carbohydrates appears to be a key aspect to be considered.

## What does “low carbohydrate” and “ketogenic” refer to?

A large number of publications refer to a diet “low in carbohydrates” or to “ketogenic”. But what levels of carbohydrate correspond to these concepts? We could start by assuming that low refers to lower than the current average intake or lower than current recommendations. According to the most recent Dutch Food Consumption Survey (FCS, 2007–2010; 7–69 years), the average daily intake of macronutrients is 45 en% from carbohydrates (of which 21 en% from sugars and 24 en% from starch), 35 en% from fats, 15 en% from protein and 15–23 g of fiber [28]. On that basis, the term "low" could refer to lower than 45 en% derived from carbohydrates. However, does that necessarily mean low in terms of the effects on our metabolism? Currently, only guidelines exist regarding the recommended daily intake of foods and there are no international guidelines on high- or low-limit values. Anything above or below the recommended intake amounts can, respectively, be referred to as high or low. Therefore, the question is which bandwidth is defined as ‘low carbohydrate’ when comparing studies to one another to draw conclusions regarding the effects. Westman [29] describes this problem as follows: “Much of the controversy when studying the outcomes of LCHF diets stems from the lack of a clear definition. The guiding principle of carbohydrate limitation is that, in response to the reduced availability of glucose and lower insulin values in the blood, the body should enter a state of increased fat burning, leading to ketosis. It would appear that a type of threshold value for carbohydrate intake exists above which this metabolic change does not clearly occur. For that reason, ketogenic studies are interpreted assuming only 20–50 g of carbohydrates per day and, if possible, a maximum of 20 g per day [29, 30]. Fewer ketone bodies are formed in the liver upon intake of more carbohydrates. In such cases, the term used is a low-carbohydrate diet rather than a ketogenic diet, the former containing 50 to max. 150 g of carbohydrates per day”.

## What are the metabolic effects of a low-carbohydrate availability?

Two processes that come into play when glucose availability is in decline: (1) gluconeogenesis, (2) ketogenesis. Below these aspects will be explained in short.

## Gluconeogenesis

**Fig. 1:**
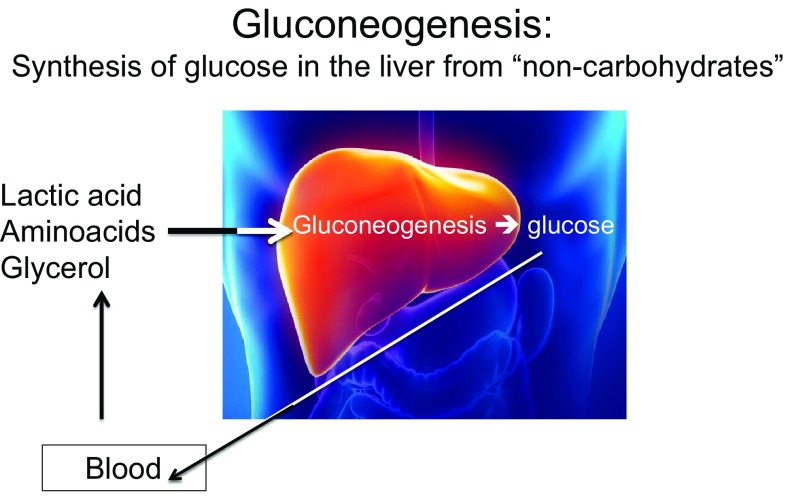
Gluconeogenesis (production of glucose) and glycolysis (breakdown of glucose) are processes that always take place simultaneously and are reciprocal (if one is high, the other is low, and vice versa). In cases of low-glucose availability from glycogen, glycolysis will be conducted at a low level and there will be a stimulus for gluconeogenesis

A very low-carbohydrate intake (< 50 g per day) will result in a decreasing glucose supply to the liver, muscles and brain, resulting in a decline in the amount of glucose stored as glycogen. When glucose availability is limiting the body will activate a process called gluconeogenesis. Gluconeogenesis (endogenous production of glucose) and glycolysis (breakdown of glucose) are processes that always take place simultaneously and are reciprocal (if one is high, the other is low, and vice versa). The primary carbon skeletons required for the synthesis of glucose in gluconeogenesis (Fig. 1) come from lactic acid, glycerol and the amino acids alanine and glutamine [31].

## Ketogenesis

**Fig. 2:**
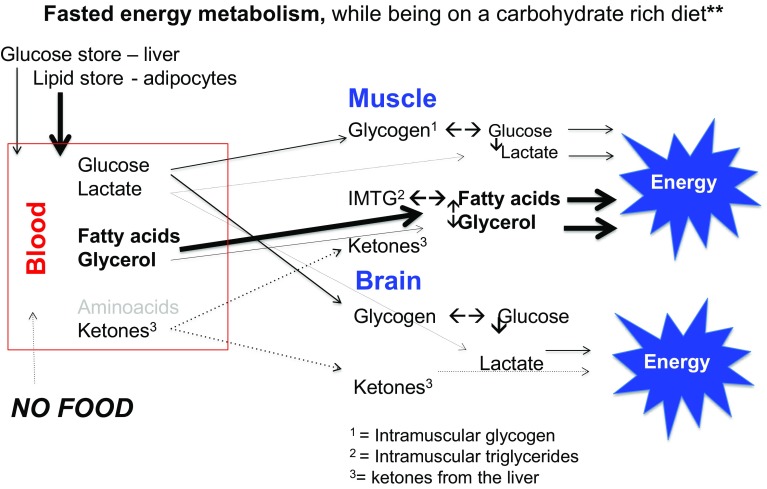
In a high-carbohydrate diet, the glucose reserves in the liver and muscles are usually well stocked. In fasting conditions, blood glucose levels are kept steady by breakdown of glucose from the liver glycogen. This is regulated by the insulin/glucagon ratio. The low insulin levels ensure that relatively few fatty acids are stored in the adipose cells, while the secretion of fatty acids by the breakdown of stored lipid (lipolysis) ensures elevated blood plasma fatty acid levels. This leads to a high degree of fatty acids oxidation and relatively low oxidation of glucose. This is then expressed in a low respiratory quotient (RQ), usually 0.75–0.8

**Fig. 3:**
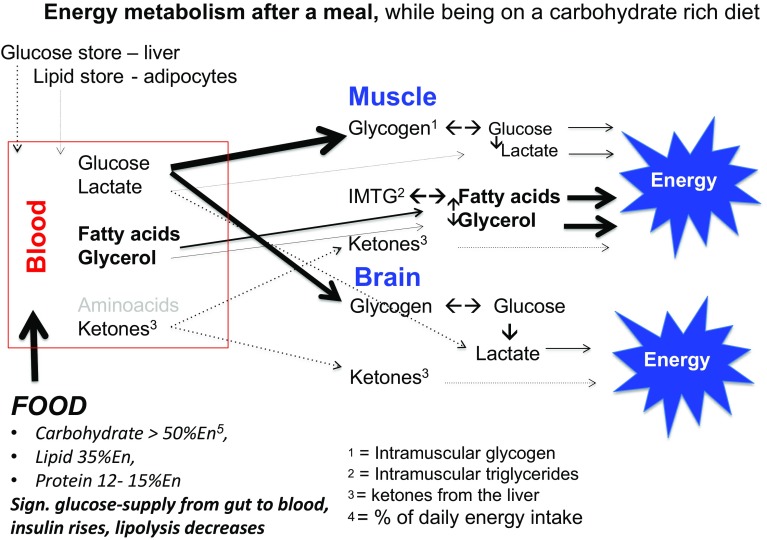
Following a carbohydrate-rich meal, the blood glucose is elevated by the supply of glucose from the intestine, resulting in elevated insulin levels and a temporary decrease in glucagon levels. This combination results in a sharp decrease in glucose production from the liver glycogen. At the same time, the release of fatty acids from the adipose cells  is inhibited and the uptake of both glucose and fatty acids from the blood is stimulated. In this case, the burning of primarily fatty acids in a fasting condition shifts to a combination of elevated glucose- and reduced fat oxidation. This is expressed in an elevated respiratory quotient (RQ), depending on the carbohydrate intake and the magnitude of the insulin response, between 0.85 and 1.0. There is also a small contribution from amino acids, which are converted into glucose via gluconeogenesis. Under normal conditions, this amounts to approx. 1–3%, although in cases of acute or chronic carbohydrate restriction resulting in significant glycogen breakdown and depending on the degree of adaptation to the situation this can even rise to > 15% [32–37]

**Fig. 4:**
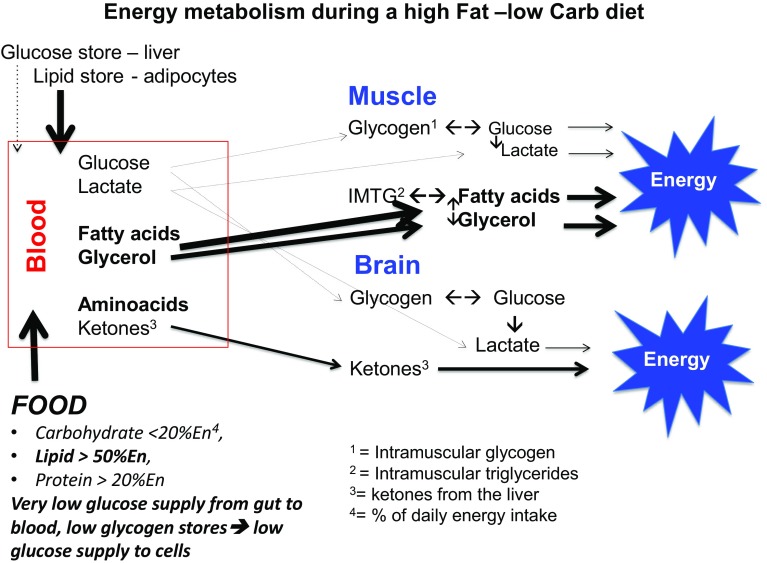
When following an LCHF diet, the amount of glucose that is taken up in the blood from the food each day is insufficient to maintain the glycogen stores in the liver and muscles. This results in an reduction of glycogen stores, reduced glucose release and consequently to reduced  blood glucose levels. The body experiences this as stress and will do everything it can to ensure it burns fatty acids as much as possible with the aim of preventing utilization of glucose, which is needed primarily for the central nervous system and the red blood cells, as much as possible. This is achieved by a sharp decrease in insulin and an increase of stress hormones. This results in an excess supply of fatty acids, leading to a partially incomplete metabolism in which ketones are produced (ketogenesis) from a part of the produced acetyl-CoA. These ketones can then be used by the brain and the muscles as an alternative fuel source instead of glucose. This is crucial to the brain, as fatty acids cannot pass through the blood–brain barrier, while glucose and ketones can. In the case of a shortage of glucose, the brain cells and neurons are able to use ketones as an alternative fuel source. There is also a small to medium contribution from amino acids, which are converted into glucose via gluconeogenesis

**Fig. 5:**
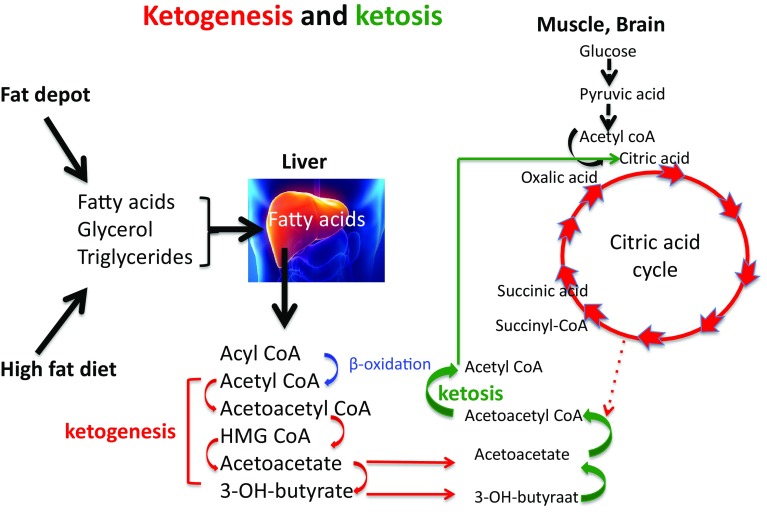
Ketogenesis is a process that takes place entirely in the liver

When endogenous production of glucose by gluconeogenesis remains too low to cover the body’s glucose needs of cells that primarily rely on glucose as a fuel, ketone bodies will be produced as an alternative to glucose [32]. In this condition, insulin levels in the blood will be low, sharply reducing the stimulus for fat and glucose storage. This observation is often referred to when promoting a high fat diet for weight maintenance and reduction of diabetes risk factors (see also further below). Other hormonal changes would subsequently lead to an increase in the breakdown of fat from the fat cells and making more fatty acids available as fuel. In this situation of a continuous elevated supply of fatty acids, not all fatty acids will be burnt completely. Acetoacetic acid (acetoacetate) is then created which is subsequently converted into the ketones beta-hydroxybutyric acid (β-hydroxybutyric acid) and acetone. For that reason, ketones are to be regarded as “a type of emergency generator that kicks in when there is a power outage”. Figures 2, 3, 4 and 5 give a schematic representation of the metabolic processes in case of a normal carbohydrate intake and after limitation of carbohydrate intake leading to ketosis.

## Do frequent carbohydrate-induced insulin responses drive overweight?

According to Hall [32], the carbohydrate–insulin model of obesity theorizes that diets high in carbohydrate are particularly fattening due to their propensity to elevate insulin secretion. Insulin directs the partitioning of energy toward storage as fat in adipose tissue and away from oxidation by metabolically active tissues and purportedly results in a perceived state of cellular internal starvation. In response, hunger and appetite increases and metabolism is suppressed, thereby promoting the positive energy balance associated with the development of obesity. Hall states that this hypothesis, which is cited by many to support recommendations for a LCHF diet, cannot be verified by controlled studies. He also suggests that the mechanisms most likely are far more complex than previously thought, given that the differences in energy consumption and body fat, as observed in the controlled case studies, are contrary to the differences that are predicted based on the carbohydrate–insulin model. Hall claims that although the rise of the prevalence of obesity may be put down to elevated consumption of refined carbohydrates, the mechanisms are most likely completely different from what we think they are. For example, consumption of foods with a high level of added sugars could result in a greater total energy intake because they have a more attractive taste, stimulate to eat more or reduce satiety. Most recently, Hall and Guo postulated that while low-carbohydrate diets have been suggested to partially subvert these processes by increasing energy expenditure and promoting fat loss, their meta-analysis of 32 controlled feeding studies, with isocaloric substitution of carbohydrate for fat, found that both energy expenditure (26 kcal/day; *P* < 0.0001) and fat loss (16 g/day; *P* < 0.0001) were greater with lower fat diets [38].

## Does LCHF diet improve insulin action?

The scientific literature shows that individuals suffering from pre-diabetes (as shown by fasting glucose and insulin levels) or suffering from diabetes and who switch to a LCHF diet soon notice a number of effects, such as weight loss, improved insulin sensitivity, fewer fluctuations in blood glucose levels and lower fasting blood glucose levels. Such changes indirectly entail reduced risks of cardiovascular diseases [13, 14, 39]. However, there are also scientists that consider these effects primarily to be the results of weight loss and not necessarily the result of a reduction in carbohydrate intake itself. Westman et al. [29] published a thematic review of the metabolic effects of LCHF diets, and concluded that LCHF diets lead to reduction of appetite and, consequently, to weight loss and corresponding improvements of various disease risk factors.

## Does LCHF reduce dietary fiber intake?

In general, most natural carbohydrate-rich food sources are high in dietary fibers and micronutrients. For that reason, the key question arises whether switching to a LCHF diet would not lead to a significant decrease in the supply fiber, known to impact negatively on gut function and overall health [40]. A study that conducted an accurate analysis of this issue and of the relationship between popular diets and food quality (expressed as a food index score) showed that diets with less than 30 en% carbohydrates ended up in the lowest index score [41, 42], indicating that there is a realistic risk of low fiber and micronutrient intakes when consuming an LCHF diet.

Based on various meta-analysis, an appropriate dietary fiber intake, for example by consuming more whole grain compared to a low intake of whole grain, is linked to a significant disease risk reductions for diabetes type 2, cardiovascular disease [43, 44], while evidence is growing that weight management may also be supported favorably [45, 46]. The fact that LCHF diets may reduce diet quality is of concern and indicates a need for carefull nutritional guidance when following such diets.

## What do meta-analyses of LCHF diets tell us?

Astrup et al. [47] initially set out to study the effects of a high-carbohydrate diet, relatively low in fat low-fat. They asserted that the effectiveness of an ad libitum diet, relatively high in carbohydrate, as was often recommended for the prevention of weight gain in patients with normal weight, or a decrease in body weight in the case of obesity, was controversial. This resulted in a meta-analysis into the effects of intervention studies which included studies in which non-diabetic individuals consumed a low-fat (thus, consequently, a relatively carbohydrate-rich) diet, a normal diet, or a diet moderately rich in fat (control group). Following a stringent selection process, the details of 16 studies were evaluated (duration of 2–12 months, 19 intervention groups, 1910 people). At the inception of the studies, the average fat consumption of the persons in the low-fat group was 37.7% (95% Cl 36.9–38.5). In the control group, this was 37.4% (36.4–38.4). Consequently, fat consumption was equal in both the low-fat and control groups. The low-fat intervention reduced fat intake in the low-fat test groups to 10.2% (8.1–12.3) while fat intake remained unchanged in the control groups. The data showed that the energy intake of the LFHC intervention groups was lower (1138 kJ/day, *P* < 00.002) and that they showed more weight loss than the control groups (3.2 kg, Cl 1.9–4.5 kg, *P* < 0.0001). The authors concluded that a reduction of the fat content in the diet, without targeted restriction of the energy intake, resulted in higher weight loss, especially in persons with the highest body weight. The foregoing corresponds to the considerations put forward by Hall et al. [48] in a publication, which bore the self-explanatory title ‘Calorie for calorie’, dietary fat restriction results in more body fat loss than carbohydrate restriction.

In 2012, Hu et al. published a meta-analysis of randomized controlled clinical studies on the effects of diets containing < 45 en% carbohydrates compared to diets that contained less than 30 en% of fat [40]. This meta-analysis also mapped the risk factors to metabolic processes. Data from 23 studies from various countries, with a total of 2788 participants (study duration of 6–24 months, including 6 studies of 15–24 months), met the inclusion criteria and were included in the analysis. Both the low-carbohydrate diet and the low-fat diet resulted in a decrease in body weight and an improvement of metabolic risk factors. The two groups showed no significant divergences in terms of decrease of body weight, waist circumference and metabolic risk factors. The authors claim that these findings suggest that low-carbohydrate and low-fat diets have similar effects on body weight reduction and related risk factors for diseases.

A recent meta-analysis by Mansoor et al. [49] concerned data of 11 randomized controlled studies (total of 1369 participants). The study revealed that participants experienced a greater decrease in body weight and of plasma triglycerides when being on a LCHF diet. However, an increase in LDL cholesterol was also observed, which is in line with the earlier observations of [50]. It has been questioned whether the overall benefits observed do outweigh that of an unfavorable LDL increase [51].

## Is a LCHF diet healthy and safe?

Many food authorities recommend relatively high-carbohydrate and high-fiber intakes as being healthy [52]. Seen in the recommendations, discussed above, the question is what the long-term health implications of LCHF diets would be, given the relatively short duration of virtually all available studies [53, 54]. The limited literature, in which LCHF studies were compared to relatively high-carbohydrate studies, does not show consistent differences in effects on body weight. The only way to get an answer to this question would be to conduct robustly controlled long-term studies (minimum of 2 years) in which carbohydrate, fat, energy and dietary fiber intake are carefully monitored along with changes in body weight. Such studies are hard to carry out and are costly and for that reason have as yet not been carried out. One study that did conduct an evaluation of this issue, and was subject to strict controls, albeit also with a relative short duration of 8 weeks [55], resulted in the conclusion that under conditions of a steady level of energy intake during a hypocaloric diet (− 500 kcal/day) a LCHF diet is just as effective as a low-fat high-carbohydrate diet. In this study weight loss was significant in both groups and improvements in insulin sensitivity were also similar. This is a strong indication that LCHF effects are primarily related to weight loss and corresponding changes to central body fat and the associated metabolic processes.

Two reviews drew the conclusion that the short-term effects of LCHF diets are positive on weight loss and blood glucose management, but also that the long-term effects have not been studied [11, 56]. The authors indicate that observed effects seemed primarily to relate to weight loss and that, for that reason, the effect of changes in the intake of carbohydrates and fats remained “unclear”. Brinkworth et al. [57] concluded that a combination of a low-carbohydrate diet combined with a restriction of energy intake would, due to reductions of fiber intake, lead to adverse effects on the quality of bowel movements and the production of short-chain fatty acids by the flora of the large intestine. They claim that it seems as if this may potentially lead to bowel disease in the long term. After a systematic review and meta-analysis Naude et al. [58] concluded that here is probably little or no difference in weight loss and changes in cardiovascular risk factors up to 2 years of follow-up when overweight and obese adults, with or without type 2 diabetes, are randomized to low CHO diets and iso-energetic-balanced weight loss diets. In a more recent review, bearing the title “Low-carbohydrate diets and type-2 diabetes: the current status of the evidence”, in the journal *Diabetes Therapy*, Dyson expressed that the state of affairs has not changed much [59]. He concluded that low-carbohydrate diets for people with type-2 diabetes could in the short-term lead to an improvement in blood glucose regulation, weight loss, and reduction of cardiovascular risk factors, but that this appeared no longer to be the case in the longer term. Overall, LCHF diets did not seem to show any superiority compared to diets with a higher carbohydrate intake. On the basis of these findings, he concludes that low-carbohydrate diets are indeed safe in the short term and are effective, but that there are no statistical differences compared with diets containing a higher carbohydrate content. For this reason, Dyson suggests that an LCHF diet should not be recommended as the standard treatment of people with type-2 diabetes [59]. This view is supported by Wyk et al. [31] who describe that “total energy intake remains the best predictor of changes in body weight. A low-carbohydrate diet, in terms of metabolic indicators and blood glucose response, does not differ a great deal from a diet with the usual amount of carbohydrates”. Very low-carbohydrate diets seems to score slightly better in this regard, but are harder to adhere to over a longer period of time (more than 6 months). Daily carbohydrate intake, for example, seemed to amount to 132–162 g per day, despite over a year of dieting and a guiding principle of less than 50 g per day. The foregoing also implies that there is still a lack of clarity regarding the long-term effects of an LCHF diet on both effectiveness as well as food safety.

The observations of Noto et al. [60] are also relevant in this regard. These authors assessed the effects of low-carbohydrate diets on probability of mortality, by way of a systematic review and a meta-analysis of the available observational studies. 17 studies were included in total, containing the data of 272,216 people of which 15,981 (5.9%) were reported dead. The results showed that the risk of mortality under conditions of LCHF diets was significantly higher. In accordance with these findings, many insiders feel that an LCHF diet should only be recommended for persons suffering from overweight and pre-diabetes or type-2 diabetes, for the reduction of bodyweight and hyperglycemia risks. They recommend that the diet only be followed under strict medical and nutritional supervision.

Regarding the question whether long-term LCHF diets may pose health risks it should also be noted that a series of recent animal experiments and human studies into the effects of an LCHF diet, for example, show adverse effects in cholesterol, homocysteine, vascular elasticity parameters have been observed during LCHF, indicating that any potential adverse long-term effects of an LCHF vascular health cannot be ruled out [29, 40, 49, 51].

In addition, adverse effects as result of high-fat exposure were reported in the following areas: brain, cognition, memory, mental well-being, Alzheimer’s, autistic behaviour [33, 34, 61–63]; obesity, metabolic dysfunction, inflammation, liver damage, cardiometabolic risks’ [34, 38, 64–68]; risks of cancer [66, 69]; osteoporosis [70]. In elegant animal models, Cani et al. [71] clearly demonstrated that high fat feeding, which induces low intakes of fermentable dietary fibers, may lead to intestinal microbiota changes which are associated with an increased intestinal permeability resulting in endotoxemia and triggers for inflammation and metabolic disorders (note: the examples are only given as an illustration, not for an exhaustive picture). These data point to possible long-term negative effects on health that should be addressed in future studies.

## LCHF for specific patient groups

Within this context, it should be noted that a Ketogenic-LCHF (KLCHF) diet is used for specific pathologies such as epilepsy and autism epilepsy and that positive effects have been documented to decrease epileptic seizures. Similarly in this instance, this is also paired with adverse side effects; in addition, the long-term effects on overall health are unknown. Following a robust Cochrane meta-analysis, for example, Martin et al. [72] concluded that randomized, controlled KLCHF studies showed promising results following application in epilepsy patients, but that the limited number of studies, small samples, and one-sided data from child populations, resulted in poor evidential quality. Within all KLCHF studies, short-term side effects were recorded such as gastrointestinal disorders, and cardiovascular complications in the longer term. For all KLCHF studies, “compliance” was a problem due to the lack of effectiveness and/or problematic diet tolerance. The authors believe that there is a lack of evidence to support a clinical application of KLCHF in adults with epilepsy.

## New insights?

A very recent overview [73] concerned randomized intervention studies, with control group, carried out between 2001 and 2015. The authors concluded as follows: a slight though significant decrease of glycated hemoglobin (HbA1c) entails a restriction of carbohydrates (CH) at all levels: 2.2% at 30 g CH/day, − 0.7% at ≤ 75 CH/day, − 1.1% at 80–90 g CH/day and − 0.9% with CH intake to 120 g/day (a logical consequence of less glucose supply to the blood, under conditions of insulin resistance) [73]. The fasting blood values and the required medication, as such, were lower which resulted in people feeling “better”. At an intake of 58% fat and 14% carbohydrates, compared with 30% fat and 53% carbohydrates, blood triglycerides decreased and HDL cholesterol increased. Decreases in body weight varied from − 8.6 to 0.9 kg, with slightly more weight loss in favor of greater carbohydrate restriction. This study, therefore, shows favorable effects in diabetes patients following a controlled LCHF diet lasting to a maximum of ≈ 2 years. To ensure the correct interpretation of this data, it should be noted that only an abstract was published and that this abstract also contains citations that lead to further questions. We must wait for the comprehensive peer reviewed publication before we can make definitive statements on this matter.

Recent work addressed the potential effectiveness of diets differing in the contents of carbohydrate and fat on weight-loss, in dependence of insulinemic and glycemic status and sheds a differentiating light on the question about which diet type may be most effective to lose weight. Hjorth et al. [74] re-evaluated the effects of diets with different glycemic loads or different fiber and whole-grain content as assessed in three large randomized trials in overweight participants (1: the DiOGenes—Diet, Obesity, and Genes study, 2: the OPUS—New Nordic Diet study and 3: the NUGENOB—Nutrient-Gene Interactions in Human Obesity-study). Effects on the concentrations of fasting plasma glucose (FPG) and fasting insulin (FI) as possible prognostic markers for successful weight loss and weight maintenance were determined. It was observed that pre-diabetic (elevated FPG) and diabetic individuals lost more weight or regained less weight when consuming a high-fat and low-carbohydrate diet than when consuming a low-fat and high-carbohydrate diet. On the contrary, in insulin-sensitive individuals, expressing normo-glycemia, beneficial effects observed were favorable when consuming a low-fat and relatively high-carbohydrate diet. Wan 2017 observed that a relatively high carbohydrate was effective for weight loss in healthy obese individuals. Based on this data, [75] concluded that disturbed insulin sensitivity and elevated FPG are important determinants for the dietary treatment of choice, being either low fat–high carbohydrate or alternatively, low carbohydrate–high fat. Accordingly, they proposed that stratifying patients for personalized dietary guidance based on pre-diet FPG outcomes may be recommendable. In another paper, however, by Snorgaard and Astrup et al. [14, 76], the same author concluded as follows: “in addition to improvements in HbA1c in the short term, there is no superiority of low-carbohydrate diets in the field of glycemic control, weight or LDL cholesterol.

Based on these observations and the seemingly conflicting conclusions, there is a need for controlled studies, “with intention to treat”, to verify these effects as a base for future evidence-based dietary recommendations.

## Is life style intervention the favorable way to go?

Based on the information presented above, it cannot be concluded that LCHF diets result in favorable effects that outweigh effects observed with less-drastic diet regimen, containing more carbohydrate quantities that are closer to daily practise.

One might argue that an improvement of the daily diet is relatively easy to achieve and also effective for disease prevention. Recent expert panels, including those of the WHO [5], the Dutch Health Council [2], the German Food Council [1], Nordic Dietary Recommendations (Scandinavian countries) [77], and the Scientific Advisory Committee on Nutrition in England [3], have concluded that diets rich in fruit, vegetables, cereals, legumes, but also moderately rich in fat and calories, combined with a sufficient amount of daily physical activity constitute the best scenario for maintaining a healthy body weight and for the prevention of chronic lifestyle diseases. This also entails moderation of (added/free) sugar intake and selecting whole-wheat products over low-fiber starch products. The quantity of fat that is unanimously recommended by all these advisory bodies is less than 40% of daily energy intake. The recommended quantity of carbohydrates for each of these advisory bodies and others [52] is over 40% of energy intake, which corresponds to more than 180 g of carbohydrates per day.

In addition to this, it is important to notice that lifestyle interventions that also focus on other factors than diet alone have been shown to result is long-term benefits. Lindström et al. [78] described long-term effects of lifestyle intervention in a Finnish population. The specific intervention goals were weight reduction (5% or more from baseline weight), dietary modification [energy proportion of total fat less than 30% and saturated fat less than 10% of total energy, dietary fiber intake 3.6 g/MJ (15 g/1000 kcal)] or more and increased physical activity (4 h per week or more). The authors showed that lifestyle intervention while being on a relative carbohydrate-rich diet in people at high risk of type 2 diabetes induces sustaining lifestyle change and results in long-term prevention of progression to type 2 diabetes. Schellenberg et al. [79] performed a meta-analysis of lifestyle programs and concluded that interventions that include exercise, dietary changes, and at least one other component are effective in decreasing the incidence of type 2 diabetes in high-risk patients, and the benefit extends beyond the active intervention phase. However, in patients who have already been diagnosed with type 2 diabetes, the evidence for benefit of comprehensive lifestyle interventions on patient-oriented outcomes was less clear.

**Key points**

1. Each type of diet that results overweight—diabetic individuals to eat less food and taking in less energy will initially result in weight loss, which in itself will lead to favorable metabolic and functional changes.
2. The available scientific literature shows that controlled diet studies (several weeks to < 2 year) with LCHF in persons with obesity and diabetes do induce favorable effects on weight loss, blood glucose and insulin as well as some less desirable effects (increase LDL cholesterol, decrease vascular reactivity).
3. Compliance with KLCHF diets appears to be poor and after some time many individuals appear to shift to higher intakes in the range of 130–160 g/day. Accordingly, targeting 100–150 g/day may be better achievable.
4. There is lack of data supporting long-term efficacy, safety and health benefits of LCHF diets. Any recommendation should be judged in this light.
5. Persons with type 2 diabetes or borderline diabetes are recommended to restrict their daily intake of rapidly digestible carbohydrates (sugars, syrups, potato, white rice, white bread, etc.). In addition, it is recommended that when switching to a diet that includes a higher portion of fat, people should primarily select products that are rich in unsaturated fatty acids.
6. Lifestyle interventions in people at high risk of developing type 2 diabetes, while maintaining a relative carbohydrate-rich diet, results in long-term prevention of progression to type 2 diabetes and are generally seen as safe.
7. Due to the complexity of the potential mechanisms, their interactions, and an absence of data from robustly controlled long-term studies (> 2 years), a general public evidence based recommendation to support KLCHF and LCHF diets as a preventive measure to help reduce risks of type 2 diabetes, seems premature. The role of long-term elevated consumption of fat combined with low-carbohydrate consumption warrants further study before general recommendations can be made.
