# Correction to: Overweight and diabetes prevention: is a low-carbohydrate–high-fat diet recommendable?

**DOI:** 10.1007/s00394-019-01959-w

**Published:** 2019-04-16

**Authors:** Fred Brouns

**Affiliations:** Department of Human biology, Faculty of Health, Medicine and Life Sciences, NUTRIM‑School of Nutrition and Translational Research in Metabolism, Maastricht University, Post Box 616, 6200 MD Maastricht, The Netherlands

## Correction to: European Journal of Nutrition (2018) 57:1301–1312 10.1007/s00394-018-1636-y

In the original publication, the disclosure of potential conflicts of interest statement was not correct.

Please find below the correct statement:

**Potential conflict of interest**

The author spoke at a meeting of the European Starch Producers Association and EU commissioners (2017) about production and potential health effects of Iso-glucose. The author contributed to the Foundation-Kenniscentrum Suiker en Voeding (Knowledge Center Sugar and Food) for 2 evidence based educational videos about sugars, metabolism and health (2017) and prepared a text entitled “Voeding met een laag koolhydraat gehalte” (Food with a low carbohydrate content) for their educational newsletter (2018). The author took part in an invited expert panel (2015–2017) convened by ILSI-EUROPE with the aim to review international nutrition and food based guidelines about sugars, carbohydrates, fibers and health published in EJCN (10.1038/s41430-017-0035-4).

